# Endodontic treatment and management of patients under antiresorptive treatment: a scoping review on MRONJ risk

**DOI:** 10.1007/s00784-025-06543-7

**Published:** 2025-09-19

**Authors:** Giulio Zavalloni, Andrea Spinelli, Martina Coppini, Rodolfo Mauceri, Giuseppina Campisi, Jacopo Lenzi, Maria Giovanna Gandolfi, Carlo Prati, Fausto Zamparini

**Affiliations:** 1 University of Bologna, via Zamboni 33, Bologna, 40126 Italy; 2 Endodontic Clinical Section, Department of Biomedical and Neuromotor Sciences, School of Dentistry, University of Bologna, Bologna, 40126 Italy; 3 Department of Precision Medicine in Medical, Surgical and Critical Care, University of Palermo, Palermo, PA Italy; 4 Department of Biomedical and Dental Sciences and Morphofunctional Imaging, University of Messina, Messina, ME Italy; 5 Unit of Oral Medicine and Dentistry for frail patients, Department of Rehabilitation, fragility, and continuity of care, Regional Center for Research and Care of MRONJ, University Hospital Palermo, Palermo, PA Italy; 6 Department of Biomedicine, Neuroscience and Advanced Diagnostics (Bi.N.D), University of Palermo, Palermo, PA Italy; 7 Department of Biomedical and Neuromotor Sciences, University of Bologna, Bologna, 40126 Italy; 8 Laboratory of Biomaterials and Oral Pathology, Department of Biomedical and Neuromotor Sciences, School of Dentistry, University of Bologna, Bologna, 40126 Italy

**Keywords:** Bisphosphonate-Associated osteonecrosis of the jaw, Endodontics, Dental education, Root canal preparation

## Abstract

**Objectives:**

To summarize the current scientific evidence on the implications of root canal treatment (RCT) in patients receiving antiresorptive therapy and to outline best practices for managing such cases to prevent MRONJ.

**Materials and methods:**

A search strategy across PubMed, Web of Science and Scopus was performed. Clinical studies and reports on RCTs in patients on antiresorptive therapy were screened by two independent reviewers. Data on patient factors (sex, age, disease, treatment, outcome) and tooth-related aspects (procedure, diagnosis, anesthesia, irrigation, obturation, antibiotics) were extracted.

**Results:**

Of the 514 studies identified, 15 articles (133 patients) met inclusion criteria. One prospective study included 65 patients and 96 RCTs; the remaining patients were reported in case reports or series and in two retrospective studies. Endodontic protocols varied largely across studies. The most common indications for RCT were pulpitis (*n* = 62) and periapical lesions with acute endodontic diseases (*n* = 119). All patients were undergoing antiresorptive therapy with oral or intravenous bisphosphonates for the management of osteoporosis or oncologic conditions. RCT was performed in all cases using conventional protocols that included local anesthesia (in some cases without vasoconstrictors), mechanical instrumentation, chemical irrigation (most commonly with sodium hypochlorite) and root canal obturation. Five studies reported antibiotic use. The cases of MRONJ (*n* = 7, 5.3%; mean treatment duration was 49 months) appeared only in oncological patients.

**Conclusions:**

RCT appears to be a safe procedure for patients receiving bisphosphonates. Cases in which RCT appeared to act as a trigger for MRONJ are rare and ambiguous and seem primarily associated with procedural errors or high-risk patients. Although some recommendations for endodontic practice to prevent MRONJ have been proposed, there is a clear need for further research in this area.

**Clinical relevance:**

Dentists can safely perform RCT in patients undergoing bisphosphonate therapy. Some clinical recommendations based on the available literature are provided.

## Introduction

### Rationale

Medication-Related Osteonecrosis of the Jaw (MRONJ) is a rare but debilitating condition, for which a consensus on classification, pathophysiology, and treatment strategies has yet to be reached [1, 2]. Originally described as Bisphosphonate-Related Osteonecrosis of the Jaw (BRONJ) by Marx et al. in 2005 [3], the condition was later redefined as MRONJ by the American Association of Oral and Maxillofacial Surgeons in 2014 to include other drug-related cases [4].

Several risk factors have been identified, particularly those associated with medications — specifically antiresorptive drugs (bisphosphonates — BPs — and denosumab) and antiangiogenic agents (e.g., bevacizumab and sunitinib) — as well as dentoalveolar procedures, especially tooth extractions [1, 2, 5]. Intravenous BPs, particularly at high doses and over prolonged periods, are most frequently implicated, often in the context of bone metastases, whereas oral BPs, commonly used for osteoporosis, rarely induce MRONJ [1]. Denosumab carries a higher MRONJ risk compared to BPs [6]. Antiangiogenic agents, though less studied, may contribute by impairing neovascularization in tumor tissues [7, 8]. Moreover, recent studies suggest that periapical inflammation may represent a more significant risk factor than the extraction procedure itself [1, 9, 10].

Despite ongoing research, no definitive pathophysiological pathway has been established. However, it is widely accepted that these medications accumulate preferentially in bones with high metabolic activity, particularly the jawbones [11]. There, they inhibit osteoclast function, reducing bone resorption, and impair vascular supply. When tissue continuity is disrupted — whether due to iatrogenic trauma (such as tooth extraction) or spontaneous events (such as periapical or periodontal infections) — bacterial invasion of the bone may occur. This often involves *Actinomyces*, a common component of the oral microbiota, and may lead to secondary infections [12, 13]. Furthermore, these areas exhibit increased bone turnover and are thus exposed to higher concentrations of medication compared to the remaining alveolar ridge [14].

Recent research has focused on dental interventions designed to lower the incidence of MRONJ among at-risk patients. Root canal treatment (RCT), as a less invasive alternative to extraction, may resolve pulpal and periapical disease and prevent microbial invasion of periodontal tissues.

Endodontic procedures should aim to preserve teeth with pulpal or apical lesions, thus delaying or avoiding extractions (“preventive endodontics”) [12, 15, 16]. Ideally, all sites of infection should be treated prior to the initiation of MRONJ-related medications [16]; however, this is not always feasible.

According to recent studies, RCTs in patients undergoing antiresorptive therapy appear to be safe, with no increased risk of MRONJ [5, 10, 17–19], although recommendations regarding the exact procedure of RCT are often lacking or contradictory. Nevertheless, RCT has also been reported as a potential MRONJ trigger [3].

An aging population and increasing medication use will likely increase the number of complex dental patients, especially with osteometabolic or oncologic conditions [20, 21]. According to Hernlund et al., approximately 5% of Europeans over the age of 50 have osteoporosis and receive BP therapy [22]. It is therefore essential for dentists to be well-prepared to manage this at-risk population effectively. Nevertheless, several studies indicate a significant lack of knowledge about MRONJ among dental professionals [23–26].

Some reviews have previously addressed the risk of MRONJ following RCT in patients undergoing antiresorptive therapy; however, these works often lacked methodological rigor and did not provide a comprehensive analysis of the suggested endodontic protocols, which often lack standardization [12, 16, 27, 28]. Given the expected variability across studies - particularly regarding the types of diseases and medications, endodontic protocols and reported outcomes - a scoping review was selected as the most suitable methodological approach. This kind of review is designed to explore the breadth of existing literature and is especially useful when addressing broad research questions. This approach allows for the inclusion of several sources of evidence without rigid eligibility criteria, thus offering a broader perspective on the topic.

### Objectives

For these reasons, the purpose of this scoping review is to map and synthesize the current clinical evidence on the relationship between treatment protocols for endodontic diseases and MRONJ appearance in patients under antiresorptive treatment and to suggest safe strategies for oral health professionals involved in the care of this at-risk population. PCC (Population/Concept/Context) framework was applied to formulate the research question [29]: “In patients undergoing antiresorptive therapy (P), what endodontic procedures and protocols (C) have been reported in the literature (C) to prevent MRONJ?”.

## Materials and methods

### Protocol and registration

This scoping review was conducted in accordance with the PRISMA Extension for Scoping Reviews (PRISMA-ScR) guidelines [29].

The review protocol was registered on the Open Science Framework (OSF) and is available at the following link: https://osf.io/x2bj8 (DOI 10.17605/OSF.IO/X2BJ8).

### Eligibility criteria

For this scoping literature review [30], the following eligibility criteria were applied: (a) full-text articles published in the English language that describe the endodontic management of human patients under antiresorptive treatment, (b) prospective studies, (c) case-reports, (d) case-series, (e) retrospective studies. The exclusion criteria were: (a) articles not published in English, (b) literature reviews, (c) letters to the editor, (d) non-peer-reviewed articles.

### Information sources

An electronic search was conducted in PubMed, Scopus, and Web of Science, covering the period from 1967 to June 2025. No additional sources of grey literature were included in the search strategy. The search strategy was developed through team discussions.

### Search

The following query, incorporating relevant terms, was used: (“endodontics” OR “root canal treatment”) AND (“BRONJ” OR “MRONJ” OR “osteonecrosis” OR “bisphosphonate” OR “denosumab”). Reference lists of the included studies were also screened to identify any additional studies not captured by electronic search.

### Selection of sources of evidence

Duplicate records from different databases were removed using Zotero (Corporation for Digital Scholarship, Vienna, USA). After a calibration exercise through test literature research, which resulted in a high level of agreement (> 90%), two reviewers (G.Z. and A.S.) independently screened all article titles and abstracts retrieved from the electronic search. In a second phase, the reviewers read the full-text manuscripts of the potentially eligible articles and removed the ones that did not meet the inclusion criteria. Any disagreements between the reviewers were solved by a third reviewer (F.Z.) who made the final decision on whether to include the studies in cases of conflict.

### Data charting process

In line with scoping review methodology, a data charting process was used to organize key study characteristics and outcomes systematically. The authors predetermined the type of data to be extracted. Two reviewers (G.Z. and A.S.) independently extracted the data and recorded it in standardized Microsoft Excel spreadsheets (Microsoft Corporation, Redmond, USA), which were later cross-checked for consistency. In some cases, the reviewers contacted the authors of the primary studies for additional information and clarification. The finalized data were then summarized in tabular form.

### Data items

Data from case reports, case series, and prospective studies are presented in Tables 1 and 2, whereas Tables 3 and 4 are dedicated to retrospective study findings.

**Table 1:**
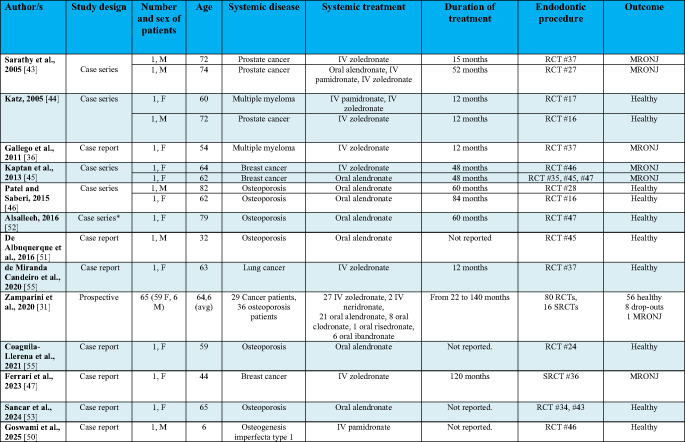
Patient-related data from case reports, case series and prospective studies. Male (M), female (F), IV (intravenous), secondary root Canal treatment (SRCT)

* Of the four patients in the original case series, two had received radiotherapy and one had undergone endodontic treatment before BP therapy, leaving only one eligible for inclusion

**Table 2:**
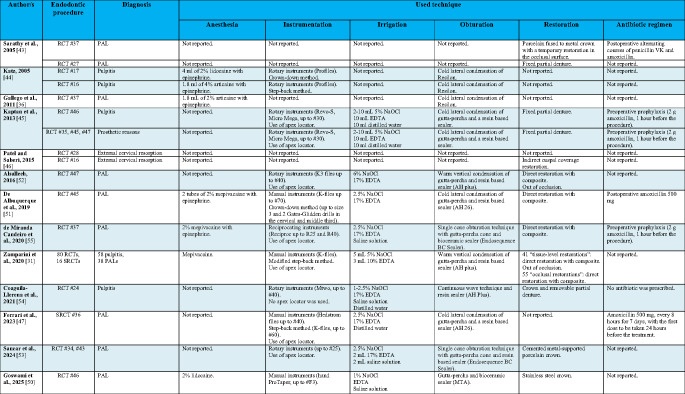
Tooth-related data from case reports, case series and prospective studies. Root Canal treatment (RCT), secondary root canal treatment (SRCT), periapical lesion (PAL)

**Table 3:** Patient-related data from retrospective studies. Male (M), female (F), IV (intravenous), primary root Canal treatment (RCT), secondary root Canal treatment (SRCT)

Author/s	Study design	Number and sex of patients	Age	Systemic disease	Systemic treatment	Duration of treatment	Endodontic procedure	Outcome
Hsiao et al., 2009 [17]	Retrospective	28, sex not reported	66,4 (avg)	Osteoporosis	Oral bisphosphonate (alendronate, risedronate, ibandronate)	60 months (avg)	26 RCTs, 8 SRCTs	Healthy
Dereci et al., 2016 [19]	Retrospective	24 (14 F, 10 M)	60,2 (avg)	Metastatic bone disease	IV zoledronate	8 patients for < 1 yr 16 patients for > 1 yr	34 RCTs, 3 SRCTs	Healthy

**Table 4:** Tooth-related data from retrospective studies. Root Canal treatment (RCT), secondary root canal treatment (SRCT), periapical lesion (PAL)

Author/s	Endodontic procedure	Diagnosis	Used technique					
			Anesthesia	Instrumentation	Irrigation	Obturation	Restoration	Antibiotic regimen
Hsiao et al., 2009 [17]	26 RCTs, 8 SRCTs	34 PALs	Not reported.	Rotary instruments. Crown-down method.	NaOCl	Warm vertical or cold lateral condensation with gutta-percha or RealSeal.	Not reported.	Not reported.
Dereci et al., 2016 [19]	34 RCTs, 3 SRCTs	37 PALs	Not reported.	Reciprocating instruments. Use of apex locator.	2.5% NaOCl 5 ml 17% EDTA 2 ml 2% chlorhexidine Additional chemicals for dissolving gutta-percha cones.	Single cone obturation technique with gutta-percha cone and resin based sealer (AH Plus).	Direct restoration with composite.	Not reported.

Tables 1 and 3 include the following patient-related data: author(s), study design, number and sex of patients, age, systemic disease, systemic treatment, duration of treatment, endodontic procedure and outcome after RCT (healthy patient or MRONJ appearance).

Tables 2 and 4 include the following tooth-related data: author(s), endodontic procedure, diagnosis and the technique used to treat the endodontic disease as described in the articles. In particular, information about the following procedures was extracted: (a) anesthesia, (b) instrumentation, (c) irrigation, (d) obturation, (e) restoration and (f) antibiotic regimen.

These items were selected to provide a comprehensive overview of both patient- and tooth-level characteristics relevant to the RCT–MRONJ relationship.

### Synthesis of results

Due to differences in study design, case reports, case series and prospective studies were analyzed separately from retrospective studies.

Data were summarized narratively and organized thematically according to phases of endodontic treatment: anesthesia, instrumentation, irrigation, obturation, restoration and antibiotic regimen.

## Results

### Selection of sources of evidence

**Fig. 1:**
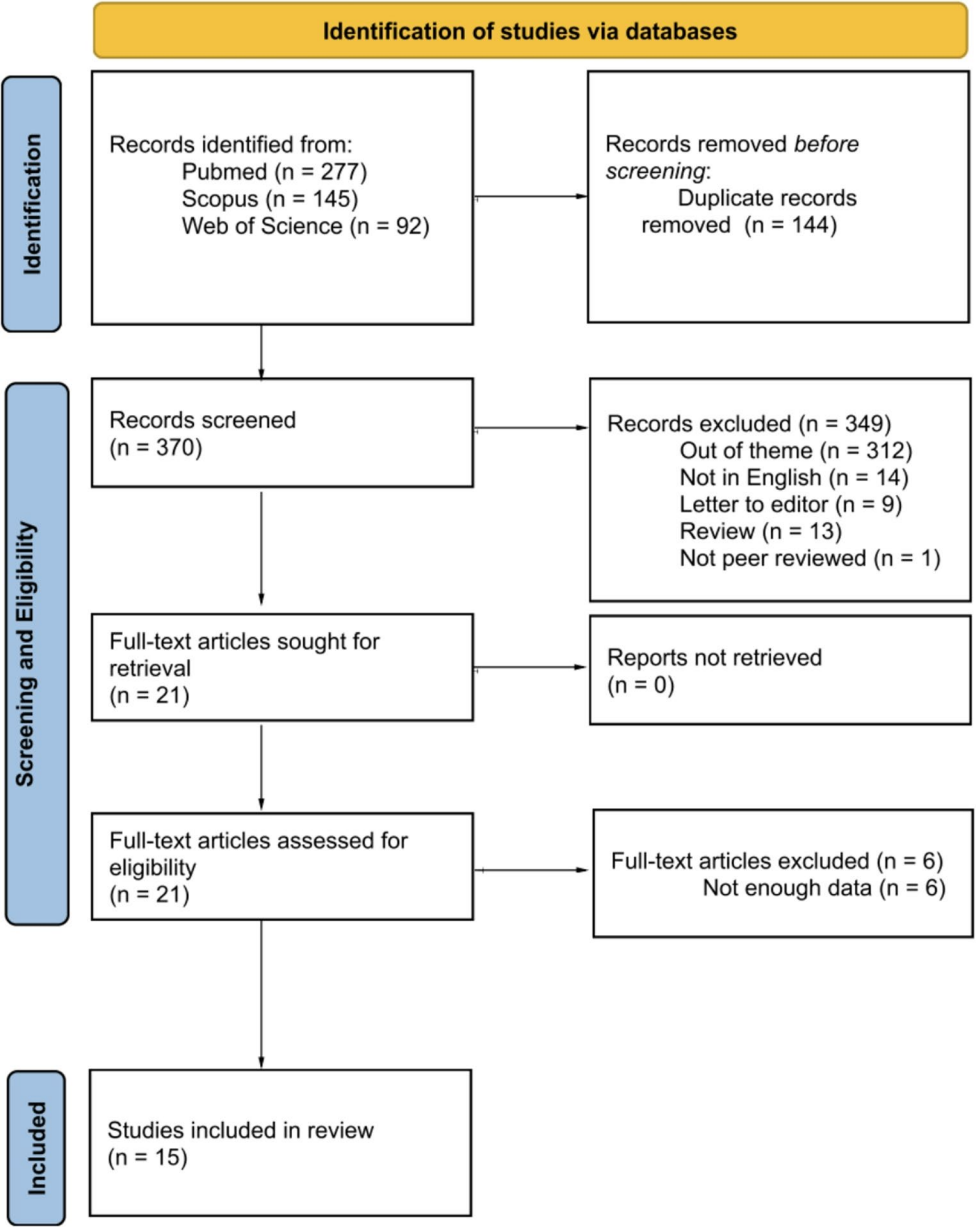
Flowchart of the search strategy used in this study

As shown in the PRISMA flowchart (Fig. 1), the electronic search identified 514 titles and abstracts. After removal of duplicates, 370 studies remained. The main reason for study exclusion at this stage was that many articles investigated the use of BPs as endodontic irrigants (e.g. etidronic acid), which was not aligned with the aim of the review and was considered out of scope. Following the screening and eligibility assessment, six additional studies were excluded due to insufficient data, which rendered them unsuitable for analysis. Only 15 studies met all the eligibility criteria. The reference lists of these studies were hand-searched; however, no additional articles were identified. The entire process of article identification, screening and inclusion is shown in Fig. 1.

### Results of individual sources of evidence

#### Case reports, case series and prospective studies

All studies were published between 2005 and 2025. Twelve out of thirteen were either case reports (*n* = 7) or case series (*n* = 5), highlighting the scarcity of high-level evidence. The only exception was a prospective clinical study with a 60-month follow-up.

The total number of patients was 81 and the number of patients per study ranged from 1 to 65 (median = 1). The retrieved studies analyzed more females than males (69 females, 12 males). The mean age was 63.6 years.

Five studies reported endodontic treatments in osteometabolic patients and six reported endodontic treatments in cancer patients. One study reported RCT in both patient groups. One study described RCT in a patient affected by a rarer disease, osteogenesis imperfecta.

All studies reported on RCT in patients being treated with BPs. No studies discussing RCT in patients treated with denosumab were retrieved. A total of 42 osteoporosis patients were being treated with oral BPs (alendronate, clodronate, risedronate, ibandronate). All case reports and series reported on patients under oral alendronate, while the biggest prospective study included also the other oral BPs. 38 cancer patients (affected by prostate cancer, breast cancer, lung cancer and multiple myeloma) were being treated with intravenous BPs (zoledronate, pamidronate, neridronate). One patient affected by osteogenesis imperfecta was being treated with intravenous pamidronate. The duration of the treatment ranged from 12 to 140 months, but was not consistently reported by the authors; therefore, an average value could not be calculated.

A total of 115 RCTs were performed. Among these, 17 were secondary root canal treatments (SRCTs), 16 of which were included in a single study. Of the endodontically treated teeth, 55 were in the mandible and 60 in the maxilla.

As shown in Table 1, a total of 7 MRONJ cases occurred after RCTs (8.6% of all patients), but it is important to note that all resolved with standard conservative or surgical treatment, with no reported mortality. Zamparini et al. reported 8 dropouts in their study [31]. RCT was found to be safe in all remaining patients (*n* = 66; 81.5%) but Zamparini et al. also reported some losses after RCT (3 teeth fractures, 3 ejected teeth, 6 periodontal losses) and incomplete healing of the periapical disease in 9 cases [31].

Endodontic treatment was performed for pulpitis in 62 (53.9%) teeth and due to periapical lesions (PALs) in 48 (41.7%) teeth. In the remaining cases, the following oral diagnoses/complaints were reported: “prosthetic reasons” in 3 teeth in a single patient and external cervical resorption in 2 teeth in 2 patients.

Three authors reported the use of mepivacaine as a local anesthetic, while two authors reported the use of both lidocaine and articaine. In seven studies, the type of local anesthetic was not explicitly specified. Two authors reported not using a vasoconstrictor in combination with the anesthetic.

Regarding the instrumentation phase, five authors reported the use of rotary files, three reported the use of manual instruments, and one reported the use of reciprocating instruments. Three authors did not explicitly state the type of instrumentation used. Six authors explicitly stated that an electronic apex locator was used, while one explicitly stated it was not used. Crown-down and step-back methods were reported to be used by different authors.

Different irrigants were used. Four authors did not report the irrigant solutions employed; all others reported the use of sodium hypochlorite (NaOCl) and ethylenediaminetetraacetic acid (EDTA). Moreover, three authors reported the use of distilled water and four reported the use of saline solution.

Regarding the obturation phase, the most commonly used technique was cold lateral condensation (five authors), followed by warm vertical condensation (two authors), single cone obturation (two authors), and continuous wave technique (one author). Gutta-percha was the most commonly used filling material (eight authors), and the most commonly reported sealer was resin-based (seven authors). Two authors reported the use of Resilon, and another two reported the use of bioceramic sealers.

A great heterogeneity among restoration techniques was noted. Four authors reported the use of a direct restoration with composite, while two recommended occlusal unloading. Six authors reported the use of different types of indirect restorations.

Regarding antibiotic treatment, not all authors explicitly reported its use. Notably, one author explicitly stated that no antibiotics were used, while five authors reported the use of different antibiotics either pre- or post-operatively (penicillin VK and amoxicillin).

#### Retrospective studies

Two retrospective studies were published in 2009 and 2016.

The total number of patients was 52: 24 in one study and 28 in the other. Just one of these studies reported the sex of the patients (14 females, 10 males). The mean age was 63.5 years.

One study reported endodontic treatments in osteometabolic patients (*n* = 28) and the other reported endodontic treatments in cancer patients (*n* = 24).

All studies reported on RCT in patients being treated with BPs. As above, no studies discussing RCT in patients treated with denosumab were retrieved. A total of 28 osteoporosis patients were being treated with oral BPs (alendronate, risedronate, ibandronate). 24 metastatic cancer patients were being treated with intravenous zoledronate. The duration of the treatment was not precisely specified but ranged from less than 12 months to up to 120 months.

A total of 71 RCTs were performed. Among these, 11 were SRCTs. Of the endodontically treated teeth, 34 were in the mandible and 37 in the maxilla.

As shown in Table 3, no MRONJ cases occurred following RCTs. Although RCT was considered safe in all cases, Hsiao et al. reported 9 instances of incomplete healing [17], while Dereci et al. observed 4 such cases, primarily in patients treated for more than one year [19].

Endodontic treatment was performed due to PALs in all 71 teeth (100%).

None of the authors reported the type of local anesthetic used.

Regarding the instrumentation phase, one author reported the use of rotary files with a crown-down technique, while another described the use of reciprocating instruments. Only one author explicitly mentioned the use of an apex locator.

NaOCl was used by both authors; however, EDTA, chlorhexidine and other chemicals for gutta-percha removal were mentioned by only one author.

In the obturation phase, one author reported using either warm vertical or cold lateral condensation with gutta-percha or RealSeal, although the sealer type was not specified [17]. The other author reported using a single cone technique with a resin-based sealer.

Only one author provided information on the type of restoration, which was a direct composite restoration.

Finally, none of the studies reported details regarding antibiotic therapy.

## Discussion

Although no definitive consensus exists regarding the pathophysiology of MRONJ, most authors agree it is a multifactorial condition in which oral infection and inflammation play key roles [32]. Antiresorptive medications inhibit osteoclast activity through different mechanisms, impairing bone remodeling. Additionally, these drugs exert antiangiogenic effects and may cause soft tissue toxicity.

Among local risk factors, procedures such as tooth extraction, placement of dental implants, ill-fitting dentures and dentoalveolar surgery are recognized as significant contributors and are generally discouraged in patients undergoing antiresorptive therapy [33, 34].

The role of endodontic therapy in patients at risk of MRONJ remains a matter of debate. While such treatment aims to manage pulpitis and PALs — both potentially associated with MRONJ through bacterial colonization of the root canal system and surrounding tissues [10] — there is no standardized protocol currently endorsed in the literature. Nowadays, RCT appears to be a safe option in many of these patients, with some studies supporting its role in reducing MRONJ risk [2, 12, 16, 18]. However, other reports have associated RCT with MRONJ onset, due to possible propagation of infection or inflammation [5, 35–38]. In particular, endodontic complications such as non-homogeneous obturation, overfilling or underfilling, root perforation, or fractures caused by improper post placement may increase the risk of osteonecrosis [35, 39].

A recent review by Pauli et al. reported that the most frequent radiographic findings in patients undergoing BP therapy were widening of the periodontal ligament space, periradicular radiolucencies and pulp calcifications [40]. However, the literature is not unanimous. For instance, one study found no significant endodontic or periapical differences in osteoporotic patients treated with BP compared to untreated osteoporotic controls [41]. Another review suggested that osteoporotic patients may have a higher prevalence of PALs than non-osteoporotic individuals [42]. This would support the notion that RCT is likely to remain a necessary intervention in this population.

Given the global increase in the population aged 65 and older — and the resulting rise in osteoporosis and cancer incidence — the use of antiresorptive and antiangiogenic agents is expected to grow, potentially leading to a greater burden of MRONJ worldwide [34]. Oral health providers should therefore be aware of current endodontic approaches that may help reduce MRONJ risk in at-risk individuals.

### Summary of evidence

In this scoping review, we aimed to analyze the current literature and provide clinical recommendations for dentists regarding the endodontic treatment of patients at risk of developing MRONJ.

In accordance with scoping review methodology, no formal appraisal of study quality was performed.

This decision reflects the nature of the included studies, most of which were case reports or case series, generally considered low-level evidence due to the absence of a control group, high risk of bias, and limited generalizability.

Due to the limited number of prospective case-control studies and the heterogeneity of outcomes and methods, a scoping review was considered the most appropriate approach. Due to the lack of comparable outcomes, only descriptive statistics were performed on the extracted variables.

We identified 15 studies—including case reports, case series, retrospective and prospective studies—published between 2005 and 2025, all addressing RCT in patients receiving BP therapy. The duration of treatment was highly variable, and the medication dosage was excluded from the analysis due to the considerable variability across studies. To date, no studies have been identified involving patients treated with denosumab who underwent RCT, likely due to the limited availability of clinical data on this specific therapeutic approach compared to BPs.

Most of the included studies were case reports or small case series. As a result, most patients derive from a single prospective study by Zamparini et al. [31] and from two retrospective studies by Hsiao et al. and Dereci et al. [17, 19].

Some early case reports and series have suggested a possible relationship between RCT and the development of MRONJ since 2005, although a definitive causal association remains unconfirmed [43–46].

Vahtsevanos et al. concluded that RCT for treating pulp and periapical diseases in these patients did not appear to affect the risk of MRONJ [18], although the endodontic techniques employed were not reported. Other studies confirmed the safety of RCT and focused on periapical healing processes. Hsiao et al. showed that osteometabolic patients receiving oral BP therapy can achieve favorable outcomes following RCT, with evidence of PAL healing and no significant differences compared to healthy controls [17]. Their study employed a crown-down instrumentation technique, followed by either warm vertical or cold lateral compaction of gutta-percha, or the use of Real Seal. In contrast, Dereci et al. found a significantly higher incidence of non-healed teeth after RCT in cancer patients treated with IV zoledronate for more than one year, using reciprocating instruments [19].

In this review, we analyzed 186 RCTs in 133 patients. Case reports, case series and prospective studies showed that a total of 115 teeth in 81 patients were treated endodontically. The most common indications for RCT were pulpitis and PALs. On the contrary, retrospective studies totaled up to 71 endodontically treated teeth in 52 patients. In this case, the only indication for RCT was the presence of PALs. Overall, PALs appeared to be a more common indication for RCT in these patients compared to pulpitis. According to Baek et al., pulp and periapical diseases are risk factors for MRONJ; therefore, regular dental check-ups are recommended before and during BP therapy to identify such conditions. Notably, performing RCT did not increase the risk of MRONJ in their study [10].

In line with the literature [1], this review confirms that MRONJ is more frequent in females (5 females vs. 2 males), although the small sample size limits the strength of this observation. This may reflect the higher use of BPs in women for osteometabolic disorders or cancer. In 6 out of 7 cases, MRONJ developed in the lower jaw, which appears to be more frequently affected by the disease. The age distribution of affected patients is also consistent with previously reported data.

Based on the reviewed studies, 5.3% of patients developed MRONJ after RCT. However, this figure must be interpreted with caution: case reports and series often focus on adverse outcomes rather than negative findings. The only large prospective study reported an incidence of 1.54% [31], which is more in line with previously published data [3] and clearly lower than the incidence of MRONJ following tooth extraction, which can be as high as 14.8% [1]. In addition, the only two studies other than case reports or case series—both retrospective— reported no incidence of MRONJ following RCT [17, 19]. Furthermore, some authors, such as Kaptan et al., have suggested that RCT may not be the primary cause of MRONJ, pointing instead to pulpal or periodontal inflammation as potential triggers, which complicates causal inference [45].

Sarathy et al. [43] reported two MRONJ cases in men over the age of 70 with diabetes and multiple medications affecting calcium metabolism, further emphasizing the multifactorial etiology. Zamparini et al. [31] did not provide detailed information on the patient who developed MRONJ, while Ferrari et al. [47] described a case occurring in a diabetic patient following a SRCT. In all MRONJ cases, patients were treated with either oral [45] or intravenous zoledronate [31, 36, 43, 45, 47] for varying durations (12 to 120 months; mean 49 months). This observation confirms the higher risk associated with zoledronate compared to other BPs [1].

All MRONJ cases were successfully managed, and no deaths were reported. These findings are consistent with previous evidence suggesting that RCT can be a safe treatment option in at-risk patients [2, 5].

As in earlier studies [1, 2], MRONJ cases in this review occurred exclusively in cancer patients and were almost entirely related to intravenous medications (6 out of 7 cases). These findings support the idea — also highlighted by Moinzadeh et al. [16] — that this subgroup warrants special attention and more cautious management.

It is noteworthy that the rate of non-healing teeth following RCT in patients undergoing BP therapy (11.83%) is comparable to the rates reported in the literature for healthy individuals [48]. Therefore, PAL healing following RCT does not appear to be significantly affected by bisphosphonate-mediated osteoclast inhibition, although further research is warranted in this area.

A high degree of heterogeneity was observed in the RCT techniques used across studies, and no standardized approach emerged. The key technical considerations and clinical recommendations derived from the literature are summarized in the following sections.

#### Anesthesia

Not all studies explicitly reported on the type of anesthetic used. Among those that did, local anesthetics with vasoconstrictors—such as lidocaine, articaine, or mepivacaine—were the most commonly administered [44, 46, 49]. Two exceptions were the studies by Zamparini et al. [31] and Goswami et al. [50], which described the use of anesthetics without vasoconstrictors. Some authors have recommended avoiding vasoconstrictors to reduce the risk of compromised blood supply, as BPs already exhibit antiangiogenic properties [16, 28]. However, this recommendation is based on theoretical concerns, and the available data suggest that the use of vasoconstrictors appears to be safe, as no MRONJ cases were attributed to their use.

Moreover, Gallego et al. observed a case of MRONJ following traumatic placement of a rubber dam clamp [36]. Therefore, minimizing periodontal trauma during treatment should be considered a priority.

#### Instrumentation

No standardized endodontic instrumentation technique emerged from the reviewed literature, as each author described different protocols. Manual [31, 47, 50, 51], rotary [17, 44, 45, 52–54], and reciprocating [19, 55] instruments were used, employing either crown-down or step-back techniques. The higher number of studies using rotary systems may reflect the fact that reciprocating systems tend to generate greater apical extrusion of debris compared to rotary ones—potentially increasing the risk of MRONJ through bacterial dissemination into the periapical area [56].

However, Lopes et al. recently demonstrated that achieving apical patency in rats treated with zoledronic acid or denosumab did not result in osteonecrosis of the jaw; it should be noted, though, that microbial contamination was not present in their model [57]. Interestingly, Goswami et al. intentionally induced apical bleeding through over-instrumentation as part of a regenerative endodontic procedure, without observing adverse outcomes [50]. Conversely, other authors have recommended avoiding apical patency to minimize the risk of extruding bacteria or irritants beyond the apex [16, 28].

The use of an electronic apex locator is particularly important to accurately identify the apical constriction and prevent extrusion of infected material from the root canal system. The apex locator has been shown to outperform radiographic methods in determining working length [58]. In line with this, seven studies reported its use [19, 31, 45, 47, 51, 52, 55]. By contrast, Coaguila-Llerena et al. described a case in which a RCT was performed without an apex locator, resulting in overinstrumentation and likely contributing to the extrusion of NaOCl; fortunately, the patient experienced no lasting complications [55].

#### Irrigation

The reviewed studies reported a wide range of irrigation protocols, using mainly NaOCl in volumes ranging from 2 mL to 10 mL and concentrations between 1% and 6%, usually in combination with EDTA (2–10 mL, 10–17%). In a few studies, saline solution [50, 53, 55, 59] and distilled water [45, 55] were also employed as irrigants.

No study reported the rationale for choosing one specific irrigant over another. Nonetheless, it would be interesting to explore this topic further. It is already well established that NaOCl can be toxic; therefore, concerns may arise in patients at risk of MRONJ [60].

Notably, Coaguila-Llerena et al. described a case of NaOCl extrusion beyond the apex, which was followed by complete remission of symptoms within 30 days [56]. While this event suggests that NaOCl extrusion may not necessarily lead to MRONJ, several factors must be considered. The patient was receiving oral alendronate rather than intravenous BPs, and the area was promptly irrigated with saline solution following the incident.

#### Obturation

The available literature reported two main types of sealers: resin-based sealers [31, 45, 47, 51–54], which were the most frequently used (96 patients; 72.2%), and premixed bioceramic sealers [50, 56]. In our opinion, future studies should further investigate the effects of different endodontic cements and the effects of their extrusion in patients at risk of MRONJ because of its possible implications. In particular, bioceramic sealers showed better biocompatibility and lower cytotoxicity compared to resin-based sealers, which have been associated with high levels of periapical and subcutaneous tissue inflammation [61].

Gutta-percha was the most commonly used core filling material, although early case reports also documented the use of Resilon [36, 44]. Only three patients were treated with Resilon, and one of them developed MRONJ; however, the author identified trauma from the rubber dam clamp as the precipitating factor [36].

As for obturation techniques, some reviews recommend approaches that minimize the risk of overfilling and overextension, in order to reduce potential periapical tissue toxicity. In this regard, cold lateral compaction is considered safer than warm techniques with respect to apical overfilling [16, 28]. Nonetheless, various techniques have been reported in the literature, including single-cone obturation [19, 53, 55] and the continuous wave technique [54]. Zamparini et al., in their prospective study, reported the safe use of warm gutta-percha compaction, with only one case of MRONJ out of 65 patients and a tooth survival rate of 85% [31]. Despite this, cold lateral condensation seems the most commonly adopted technique overall [17, 36, 44, 45, 47, 51].

#### Restoration

Following RCT, both direct [19, 31, 51, 52, 55] and indirect [43, 45, 46, 50, 53, 54] restoration techniques have been reported. Some authors also suggested that occlusal-unloaded crowns may represent a safe post-endodontic option to minimize mechanical stress and reduce the risk of tooth fractures [28, 31]. This approach could help prevent the need for extractions—a high-risk procedure in patients undergoing antiresorptive therapy [28, 31, 52].

#### Antibiotic regimen

The final aspect considered in this review was the antibiotic regimen. According to Moinzadeh et al., there is currently no evidence supporting the prophylactic use of antibiotics in patients treated with BPs prior to RCT [16]. Most of the included studies did not report whether antibiotics were used; only one study explicitly stated that no antibiotic was prescribed [54], while others reported the administration of amoxicillin pre- or post-operatively [43, 45, 47, 51, 55]. The widespread use of antibiotics despite the lack of clinical evidence may stem from medicolegal considerations [16, 59].

A review by Segura-Egea et al. indicated that the risk of infection with subsequent osteonecrosis is substantially higher in cancer patients receiving BPs than in those treated for osteoporosis. The risk is further increased in cases of concomitant glucocorticoid therapy, older age, poorly controlled diabetes, intravenous administration and prolonged BP use [59]. The authors recommend a comprehensive medical evaluation on a case-by-case basis, weighing the risk of infection-related complications against the potential adverse effects of antibiotics.

Finally, pain and radiological signs of MRONJ may mimic those of odontogenic lesions and should therefore be included in the differential diagnosis [36, 62]. A thorough review of the patient’s medical history, clinical vitality testing, and awareness of drug-induced lesion patterns are essential for an accurate diagnosis and appropriate treatment.

### Limitations

This scoping review presents several limitations. First, only studies published in English and indexed in three major databases were included, which may have led to the omission of relevant data.

There is substantial heterogeneity among the included studies in terms of patient characteristics, medication types and dosages, duration of the treatments, comorbidities, endodontic techniques, and outcome measures. These data are often lacking or inconsistently reported. Moreover, the evidence base consists predominantly of case series and case reports, with only one prospective and two retrospective studies available. The low incidence of MRONJ, along with the reduced life expectancy and clinical complexity of many cancer patients, hinders the feasibility of large, high-quality trials to support evidence-based recommendations [16]. In this context, the protocol proposed by Zamparini et al., as the study with the largest number of patients, may currently offer the most reliable reference [31].

Important clinical variables—such as systemic conditions, concomitant drug use, smoking, periodontal status, denture use and oral hygiene—were inconsistently reported and therefore not systematically analyzed, although they may act as potential confounders in the association between RCT and MRONJ.

## Conclusion

This scoping review summarizes the current evidence on the relationship between endodontic treatments and the risk of MRONJ in patients receiving BP therapy. Overall, RCT appears to be a safe therapeutic option, although isolated case reports and case series have suggested a potential association with MRONJ onset, particularly in high-risk patients. MRONJ following RCT is, however, rare, with a reported incidence of 5.3%, though case reports and case series may overemphasize such occurrences.

The clinical considerations outlined in the discussion are intended to support decision-making in complex cases. However, the available evidence remains limited in both quantity and quality. Well-designed prospective studies are needed to clarify the causal role, if any, of endodontic treatment in MRONJ development and to establish standardized treatment protocols for this patient population.

## Data Availability

No datasets were generated or analysed during the current study.
